# Treatment planning comparison of volumetric modulated arc therapy with the trilogy and the Halcyon for bilateral breast cancer

**DOI:** 10.1186/s13014-021-01763-z

**Published:** 2021-02-18

**Authors:** Tao Sun, Xiutong Lin, Guifang Zhang, Qingtao Qiu, Chengqiang Li, Yong Yin

**Affiliations:** Department of Radiation Physics and Technology, Shandong Cancer Hospital and Institute, Shandong First Medical University and Shandong Academy of Medical Sciences, 440 Jiyan Road, Jinan, 250117 Shandong China

**Keywords:** Bilateral breast cancer, Volumetric modulated arc therapy, Halcyon, Trilogy, Dosimetry

## Abstract

**Background:**

The Halcyon is a new machine from the Varian company. The purpose of this study was to evaluate the dosimetry of the Halcyon in treatment of bilateral breast cancer with volumetric modulated arc therapy.

**Methods:**

On CT images of 10 patients with bilateral breast cancer, four Halcyon plans with different setup fields were generated, and dosimetric comparisons using Bonferroni’s multiple comparisons test were conducted among the four plans. Whole and partial arc plans on the Trilogy and the Halcyon, referred to as T-4arc, T-8arc, H-4arc and H-8arc, were designed. The prescription dose was 50 Gy in 2-Gy fractions. All plans were designed with the Eclipse version 15.5 treatment planning system. The dosimetric differences between whole and partial arc plans in the same accelerator were compared using the Mann–Whitney U test. The better Halcyon plan was selected for the further dosimetric comparison of the plan quality and delivery efficiency between the Trilogy and the Halcyon.

**Results:**

Halcyon plans with high‐quality megavoltage cone beam CT setup fields increased the D_mean_, D_2_ and V_107_ of the planning target volume (PTV) and the V_5_ and D_mean_ of the heart, left ventricle (LV) and lungs compared with other Halcyon setup plans. The mean dose and low dose volume of the heart, lungs and liver were significantly decreased in T-8arc plans compared to T-4arc plans. In terms of the V_5_, V_20_, V_30_, V_40_ and D_mean_ of the heart, the V_20_, V_30_, V_40_ and D_mean_ of the LV, the V_30_, V_40_, D_max_ and D_mean_ of the left anterior descending artery (LAD), and the V_5_ and V_40_ of lungs, H-8arc was significantly higher than H-4arc (*p* < 0.05). Compared with the Trilogy’s plans, the Halcyon’s plans reduced the high-dose volume of the heart and LV but increased the mean dose of the heart. For the dose of the LAD and the V_20_ and V_30_ of lungs, there was no significant difference between the two accelerators. Compared with the Trilogy, plans on the Halcyon significantly increased the skin dose but also significantly reduced the delivery time.

**Conclusion:**

For the Halcyon, the whole-arc plans have more dosimetric advantages than partial-arc plans in bilateral breast cancer radiotherapy. Although the mean dose of the heart and the skin dose are increased, the doses of the cardiac substructure and other OARs are comparable to the Trilogy, and the delivery time is significantly reduced.

Breast cancer is one of the most common malignant tumours in women. Postoperative radiotherapy can significantly reduce the recurrence rate of tumours, reduce mortality, and prolong the survival time of patients with breast cancer [1]. Bilateral breast cancer is relatively rare, accounting for approximately 2.1% of all patients with breast cancer [2]. Because of the larger target area and more complex shape, it is more difficult to design the treatment plan for bilateral breast cancer.

The Halcyon linear accelerator (LINAC) is a new machine from Varian company that offers a single 6 MV flattening-filter-free (FFF) X-ray with a jawless design. The Halcyon has many differences from conventional C‐arm LINACs, such as jawless design, a dual-layer multi-leaf collimator (MLC), faster gantry rotation and obligatory daily image-guided radiotherapy (IGRT). Comparable dose distribution and excellent delivery efficiency have been shown on the Halcyon in radiotherapy for head and neck, brain, unilateral breast, and cervical cancers [3–7]. Trilogy is a conventional C-arm LINAC with jaw and Millennium MLC.

It has been proven that volumetric modulated arc therapy (VMAT) can improve the quality of the plan and the delivery efficiency in bilateral breast cancer [8–10]. A previous study found that the partial-arc VMAT plans significantly reduced the cardiopulmonary dose compared with the whole-arc VMAT plans in unilateral breast radiotherapy [11]. Similarly, partial-arc VMAT plans were feasible in bilateral breast radiotherapy [12]. These studies were based on conventional C-arm LINAC with jaw. To our knowledge, applications of the Halcyon in the VMAT plans for bilateral breast cancer have not been reported. It is unknown whether the partial-arc VMAT plan is applicable on the Halcyon. With the Halcyon 1.0, high-quality/low-dose megavoltage cone beam computed tomography (MV CBCT) or orthogonal MV radiograph pair could be selected for image guidance, and the four setup-fields delivered different monitor units (MUs).

The aim of this study was to compare the dosimetric differences of the four setup-field plans and the suitable arc modes of VMAT plans for the Halcyon in bilateral breast radiotherapy and to analyse the plan quality and delivery efficiency by comparing the dosimetric differences between the Halcyon and the Trilogy to guide the clinical application in bilateral breast radiotherapy.

## Materials and methods

### Patient selection and volume delineation

From September 2006 to December 2018, CT image datasets of 10 patients diagnosed with bilateral breast cancer and who received bilateral breast radiotherapy at Shandong Cancer Hospital were selected. The clinical target volume (CTV) included all bilateral breast tissue, excluding local lymph node region. The planning target volume (PTV) was generated by expanding a 5-mm margin from the CTV and was shrunk to 5 mm below the skin on the skin side. The organs at risk (OARs) include the total lung, heart, left ventricle (LV), left anterior descending artery (LAD) and liver. The skin is defined as the 3-mm region below the body outside of the PTV. The normal structures were defined as the body minus the PTV (B-P).

### Treatment planning

With the Halcyon version 1.0, all imaging setup fields are taken using digital megavoltage imaging panels. When designing a Halcyon plan, four different setup fields can be selected. For the 10 patients, we designed four VMAT plans with different setup fields on the Halcyon: high‐quality MV CBCT (the gantry rotates clockwise from 260° to 100°, delivering 10 MUs, simply called CBCT-H); low‐dose MV CBCT (delivering 5 MUs in a clockwise gantry rotation from 260° to 100°, simply called CBCT-L); high‐quality orthogonal MV radiograph pair (images acquired with 0° and 90° and delivering 2 MUs for each field, simply called MV-H) and low‐dose orthogonal MV radiograph pair (images acquired with 0° and 90° and delivering 1 MU for each field, simply called MV-L). For the four plans, two anticlockwise 160°–200°and two clockwise 200°–160° rotation arcs were used.

On the Trilogy, whole and partial arc plans, referred to as T-4arc and T-8arc, respectively, were generated. The whole-arc plan consisted of two anticlockwise 160°–200° and two clockwise 200°–160° rotation arcs. The partial-arc plan consisted of total 8 partial arcs. For unilateral breast, four 100° arcs like a bowknot were generated. In the two plans, the medial x-jaw was set to the minimum site (− 2 cm) to minimize the irradiated volume of the lungs and heart. A 6 MV X-ray was used, and the dose rate was set to 600 MU/min. Two whole and partial arc plans, referred to as H-4arc and H-8arc, were designed on the Halcyon with the same arc angle as T-4arc and T-8arc mentioned above. In the Halcyon plans, a 6 MV FFF X-ray was used at the maximum dose rate of 800 MU/min. Low-dose MV CBCT was selected for image guidance as Flores-Martinez et al. [6] suggested.

The prescription dose was 50 Gy in 2-Gy fractions. All plans were designed with the Eclipse version 15.5 treatment planning system (Varian Medical Systems, Palo Alto, CA, USA) using an analytic anisotropic algorithm (AAA). For all VMAT plans, the PTV was extended to 5 mm outside the skin, named PTVop, a 10-mm bolus was used on the skin outside the PTV, and the dose of PTVop was optimized. In the final dose calculation, the bolus was deleted. The dose normalization was 95% volume of the PTV received 100% prescription dose. All plans used the same optimization parameter settings, with the goal of minimizing the doses to the lungs, heart and LAD while ensuring PTV dose coverage. No dose constraints were applied to the skin and LV during the optimization.

### Dosimetric evaluation

The dose statistics of the plans were based on dose-volume histogram (DVH) analysis. For PTV, the dose of 2% and 98% volume (D_2_, D_98_), the volume receiving 107% and 110% of the prescribed dose (V_107_ and V_110_) and the mean dose (D_mean_) were analysed. The conformity index (CI) and the homogeneity index (HI) of the PTV were calculated according to the following formula:

$$CI=\frac{{{TV}_{PV}}^{2}}{TV\times PV}$$

TV_PV_ represents the volume of the PTV wrapped by the prescription dose, the TV represents the volume of the PTV, and the PV represents the total volume wrapped by the prescription dose. Larger CI values indicate the better conformity of the target [13].

$$HI=\frac{{D}_{2}-{D}_{98}}{{D}_{p}}\times 100{\%}$$

D_p_ represents the prescribed dose. Lower HI values indicate the better uniformity of the target [14].

For OARs, the V_X_ and mean doses were analysed. V_X_ represents the irradiated volume of X Gy dose.

The number of MUs was analysed for all plans. The delivery times of T-4arc, T-8arc, H-4arc and H-8arc were recorded. The delivery time was recorded from the first field beam on to the last field beam off, excluding the positioning time.

### Statistical analysis

All data were statistically analysed using the Statistical Package for Social Sciences v20.0 software (SPSS Inc., Chicago, IL, USA). First, a one-way analysis of variance (ANOVA) using Bonferroni’s multiple comparisons test was applied to compare the four different setup-field plans on the Halcyon. Second, whole and partial arc plans on the same LINAC were compared to determine the most suitable field mode for the machine. The Mann–Whitney U test was used. The better Halcyon plan was selected for further comparisons. Third, a statistical comparison of the better Halcyon plan and the Trilogy plan was implemented to analyse the dosimetric differences between the two machines using the Mann–Whitney U test. The differences were considered statistically significant when *p* < 0.05.

## Results

### Dose comparisons for different setup-field plans

Table 1 shows the dose parameters for the four different setup-field plans on the Halcyon. Among the four plans, no significant differences were observed in the D_98_, V_110_, CI and HI of the PTV. Compared to CBCT-L, CBCT-H plans increased the D_mean_, D_2_ and V_107_ of the PTV. For OARs, CBCT-H plans increased the V_5_ and D_mean_ of the heart, LV and lungs, V_5_ of LAD and B-P. For the skin, liver and MUs, there were no significant differences between the four plans. No significant differences were found among any of the dosimetric indicators of the PTV and OARs in the CBCT-L, MV-H and MV-L plans. CBCT-L plans showed the lowest values in the mean dose of heart, LAD, LV, lungs, and liver compared to MV-H and MV-L plans, but it was not statistically significant.

**Table 1:** Dosimetric analysis for four different setup-field plans on the Halcyon

	CBCT-H	CBCT-L	MV-H	MV-L	*p*	*p* < 0.05
PTV						
D_mean_(Gy)	52.4 ± 0.3	52.0 ± 0.2	52.2 ± 0.2	52.1 ± 0.3	0.01	a
D_98_(Gy)	48.5 ± 0.2	48.5 ± 0.3	48.5 ± 0.3	48.6 ± 0.4	0.95	
D_2_(Gy)	54.3 ± 0.60	53.6 ± 0.3	53.8 ± 0.4	53.7 ± 0.5	0.01	a,c,
V_107_ (%)	16.1 ± 12.8	3.6 ± 2.8	7.9 ± 6.7	7.5 ± 10.8	0.03	a
V_110_ (%)	0.8 ± 1.6	0.0 ± 0.0	0.1 ± 0.1	0.0 ± 0.1	0.09	
CI	0.86 ± 0.02	0.86 ± 0.02	0.86 ± 0.02	0.86 ± 0.02	0.96	
HI	11.6 ± 1.3	10.1 ± 1.1	10.6 ± 1.1	10.1 ± 1.4	0.06	
Heart						
V_5_(%)	55.0 ± 9.3	30.5 ± 6.2	33.2 ± 8.4	29.4 ± 7.7	0.00	a,b,c
V_30_(%)	1.1 ± 0.6	0.8 ± 0.5	1.0 ± 0.7	1.1 ± 0.7	0.62	
D_mean_(Gy)	7.1 ± 0.7	5.6 ± 0.6	6.0 ± 0.9	5.7 ± 0.9	0.00	a,b,c
LV						
V_5_(%)	57.9 ± 11.2	35.7 ± 9.3	39.9 ± 11.6	36.0 ± 10.4	0.00	a,b,c
V_30_(%)	2.6 ± 1.3	1.9 ± 1.0	2.5 ± 1.5	2.6 ± 1.5	0.56	
D_mean_(Gy)	8.7 ± 1.3	6.8 ± 1.1	7.3 ± 1.5	6.9 ± 1.4	0.03	a,c
LAD						
V_5_(%)	97.5 ± 5.1	84.6 ± 13.4	85.1 ± 12.7	80.6 ± 14.5	0.02	c
V_30_(%)	21.3 ± 13.8	14.3 ± 10.9	20.8 ± 15.0	21.6 ± 15.0	0.59	
D_max_(Gy)	39.5 ± 3.2	38.9 ± 3.1	39.7 ± 3.3	39.8 ± 3.5	0.92	
D_mean_(Gy)	19.2 ± 4.7	17.2 ± 4.0	18.5 ± 4.6	18.2 ± 5.0	0.80	
Lungs						
V_5_(%)	57.7 ± 8.9	43.7 ± 4.1	46.6 ± 7.4	43.7 ± 6.6	0.00	a,b,c
V_20_(%)	14.9 ± 2.9	13.2 ± 2.5	14.7 ± 2.7	14.2 ± 2.4	0.47	
D_mean_(Gy)	10.4 ± 1.1	9.1 ± 0.8	9.6 ± 1.0	9.3 ± 0.9	0.03	a
Liver						
D_mean_(Gy)	6.9 ± 1.5	6.0 ± 1.1	6.5 ± 1.5	6.3 ± 1.4	0.54	
Skin						
V_40_(%)	41.3 ± 9.2	40.4 ± 8.8	39.7 ± 9.2	39.2 ± 8.5	0.96	
V_50_(%)	0.3 ± 0.2	0.3 ± 0.2	0.1 ± 0.1	0.1 ± 0.1	0.12	
B-P						
V_5_(%)	32.9 ± 4.5	27.6 ± 2.8	28.9 ± 3.5	28.0 ± 3.4	0.01	a,c
V_10_(%)	19.9 ± 2.3	18.4 ± 1.9	19.4 ± 2.0	19.0 ± 2.1	0.44	
MUs	936.5 ± 68.2	1036.1 ± 103.8	956.6 ± 73.4	986.2 ± 76.5	0.05	

a: CBCT-H versus CBCT-L; b: CBCT-H versus MV-H; c: CBCT-H versus MV-L; d: CBCT-L versus MV-H; e: CBCT-L versus MV-L; f: MV-H versus MV-L

### Dose comparisons for different arc plans on identical LINACs

Table 2 shows the PTV dosimetric parameters of the four whole and partial arc plans on the Halcyon and Trilogy LINACs. For PTV, there were no statistically significant differences in D_mean_, D_2_, D_98_, V_107_, V_110_ and HI between whole and partial arc plans on the Trilogy (*p* < 0.05). T-4arc showed better conformity than T-8arc (*p* < 0.05). On the Halcyon, the D_2_, V_107_, V_110_ and HI of the PTV in H-8arc were higher than those in H-4arc (*p* < 0.05). The D_98_ and CI in H-4arc plans were higher than those in H-8arc plans, and the differences were statistically significant. There was no significant difference in the D_mean_ of the PTV between the two plans on the Halcyon.

**Table 2:** Dosimetric parameters of PTV for whole and partial arc plans on the Trilogy and Halcyon

PTV	T-4arc	T-8arc	H-4arc	H-8arc	*P* < 0.05
D_mean_(Gy)	51.8 ± 0.2	51.6 ± 0.3	52.0 ± 0.2	52.2 ± 0. 2	c,d
D_2_(Gy)	53.5 ± 0.4	53.1 ± 0.6	53.6 ± 0.31	54.2 ± 0.47	b,c
D_98_(Gy)	48.4 ± 0.4	48.0 ± 0.6	48.5 ± 0.3	48.1 ± 0. 4	b,c
V_107_(%)	3.2 ± 2.4	1.4 ± 2.2	3.6 ± 2.8	11.1 ± 8.4	b,c
V_110_(%)	0.0 ± 0.1	0.0 ± 0.0	0.0 ± 0.0	0.4 ± 0.5	b
CI	0.87 ± 0.02	0.82 ± 0.04	0.86 ± 0.02	0.83 ± 0.03	a,b,c
HI(%)	10.3 ± 1.5	10.2 ± 2.1	10.1 ± 1.1	12.0 ± 1.4	b

a: T-4arc versus T-8arc, b: H-4arc versus H-8arc, c: T-8arc versus H-4arc, d: T-4arc versus H-4arc

**Fig. 1:**
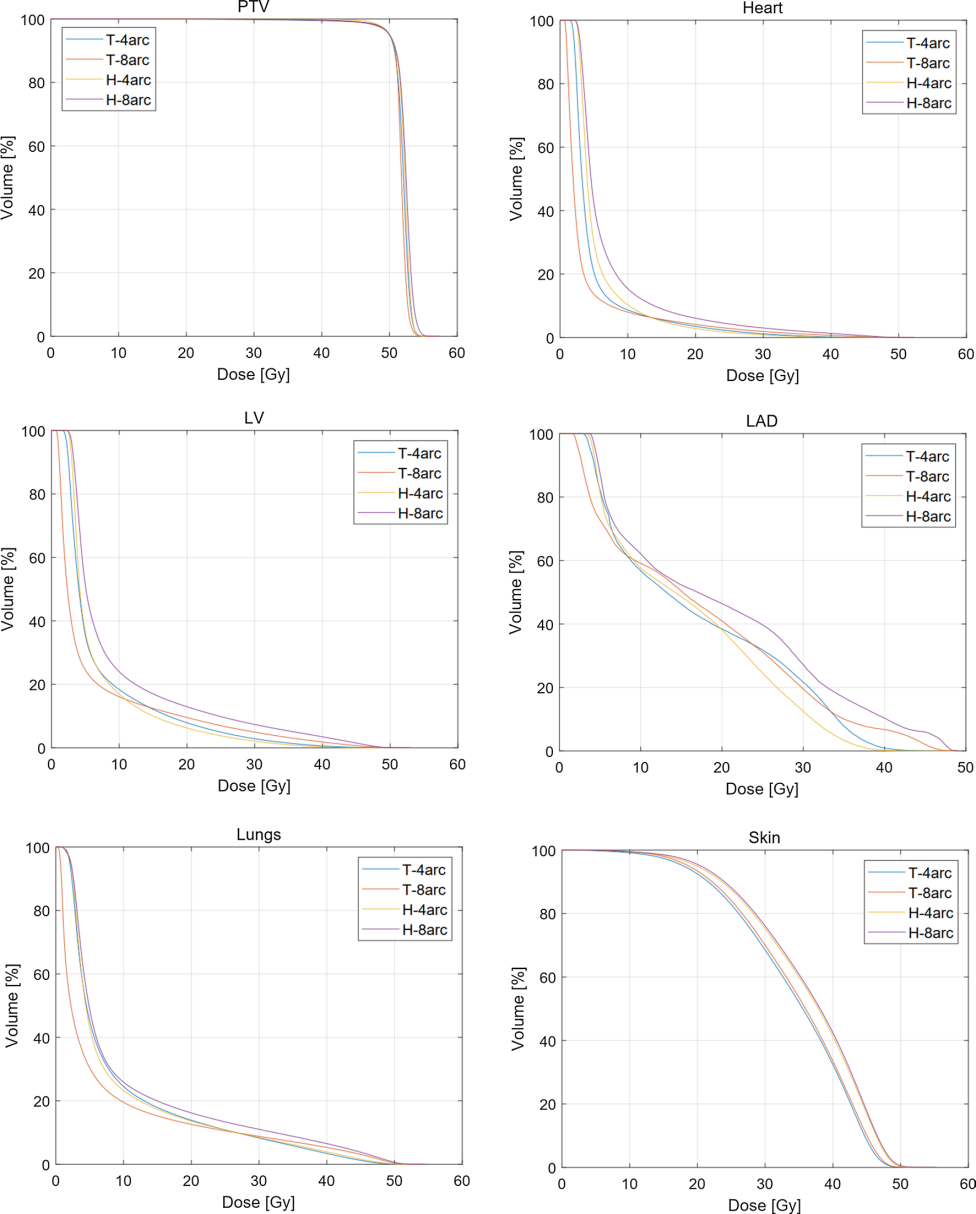
Mean dose-volume histograms for the four plans

Table 3 shows the dosimetric parameters of OARs. On the Trilogy, T-8arc plans significantly reduced the V_5_ and D_mean_ of the heart, the V_5_ of LV, V_5_, V_10_ and D_mean_ of the lungs, the D_mean_ of the liver and total MUs compared with T-4arc. T-4arc plans reduced the V_40_ of the LV and lungs compared with T-8arc. No significant differences were observed in the doses of the LAD and skin and the delivery time. On the Halcyon, lung doses (V_5_ and V_40_), heart doses (V_5_, V_20_, V_30_, V_40_ and D_mean_), and LV (V_20_, V_30_, V_40_ and D_mean_) and LAD (V_30_, V_40_, D_max_, and D_mean_) doses were significantly reduced in 4arc plans compared with 8arc plans (*p* < 0.05). H-8arc plans significantly reduced the number of MUs compared with H-4arc plans. No significant differences were observed in the skin dose and the delivery time. Figure 1 shows the mean dose-volume histograms for the four plans.

**Table 3:** Dosimetric parameters of OARs and delivery efficiency for whole and partial arc plans on the Trilogy and Halcyon

	T-4arc	T-8arc	H-4arc	H-8arc	*p* < 0.05
Heart					
V_5_ (%)	20.4 ± 6.2	13.7 ± 4.9	30.5 ± 6.2	49.2 ± 4.5	a,b,c,d
V_10_ (%)	8.5 ± 3.3	8.0 ± 3.7	10.2 ± 2.9	15.0 ± 6.4	–
V_20_ (%)	3.4 ± 1.5	4.0 ± 2.4	2.7 ± 1.3	5.8 ± 4.0	b
V_30_ (%)	1.1 ± 0.6	1.8 ± 1.7	0.8 ± 0.5	2.7 ± 2.8	b,c
V_40_ (%)	0.2 ± 0.2	0.6 ± 0.9	0.1 ± 0.1	1.1 ± 1.8	b,c
D_mean_ (Gy)	4.9 ± 0.6	3.9 ± 1.1	5.6 ± 0.6	7.1 ± 1.6	a,b,c,d
LV					
V_5_ (%)	34.2 ± 10.3	23.9 ± 7.6	35.7 ± 9.3	48.9 ± 14.8	a,c
V_10_ (%)	17.5 ± 6.6	15.5 ± 6.6	16.1 ± 5.9	22.9 ± 9.8	–
V_20_ (%)	7.6 ± 3.0	9.1 ± 5.2	5.8 ± 2.7	12.0 ± 7.7	b
V_30_ (%)	2.7 ± 1.3	4.7 ± 4.0	1.9 ± 1.0	6.5 ± 6.2	b,c
V_40_ (%)	0.5 ± 0.5	1.6 ± 2.0	0.2 ± 0.1	2.9 ± 4.1	a,b,c
D_mean_ (Gy)	6.9 ± 1.3	6.0 ± 2.0	6.8 ± 1.1	9.2 ± 3.0	b
LAD					
V_5_ (%)	85.7 ± 14.5	76.0 ± 11.4	84.6 ± 13.4	89.3 ± 8.7	–
V_10_ (%)	61.6 ± 16.5	63.6 ± 16.9	63.2 ± 17.2	67.5 ± 16.6	–
V_20_ (%)	43.3 ± 17.7	47.5 ± 21.8	43.1 ± 19.1	52.9 ± 21.7	–
V_30_ (%)	24.2 ± 15.5	25.7 ± 20.5	14.3 ± 10.9	31.2 ± 18.2	b
V_40_ (%)	1.2 ± 1.5	6.5 ± 15.4	0.1 ± 0.3	9.8 ± 15.9	b
D_max_ (Gy)	40.4 ± 4.2	41.9 ± 4.2	38.9 ± 3.1	42.5 ± 4.4	b
D_mean_ (Gy)	18.1 ± 4.7	19.0 ± 6.3	17.2 ± 4.0	21.3 ± 5.8	b
Lungs					
V_5_ (%)	45.1 ± 3.1	30.4 ± 3.5	43.7 ± 4.1	49.1 ± 4.7	a,b,c
V_10_ (%)	23.9 ± 2.6	19.4 ± 2.6	22.9 ± 2.6	25.5 ± 2.9	a,c
V_20_ (%)	13.4 ± 2.2	12.4 ± 2.6	13.2 ± 2.5	15.8 ± 3.0	–
V_30_ (%)	7.9 ± 1.8	8.5 ± 2.3	8.0 ± 2.1	10.6 ± 3.0	–
V_40_ (%)	3.2 ± 1.1	5.0 ± 1.9	3.5 ± 1.4	6.0 ± 2.5	a,b
D_mean_ (Gy)	9.1 ± 0.7	7.5 ± 1.0	9.1 ± 0.8	9.2 ± 3.0	a,c
Skin					
V_30_ (%)	67.3 ± 8.7	68.9 ± 8.2	74.2 ± 7.6	75.2 ± 6.9	c,d
V_40_ (%)	31.3 ± 7.9	32.4 ± 8.5	40.4 ± 8.8	41.5 ± 7.3	c,d
V_45_ (%)	7.9 ± 3.7	9.3 ± 3.7	16.8 ± 4.3	17.5 ± 3.3	c,d
V_50_ (%)	0.0 ± 0.1	0.0 ± 0.0	0.3 ± 0.2	0.5 ± 0.5	c,d
D_mean_ (Gy)	33.9 ± 2.0	34.3 ± 1.9	36.0 ± 1.9	36.3 ± 1.6	c,d
Liver					
D_mean_ (Gy)	5.7 ± 1.5	3.9 ± 1.1	6.0 ± 1.1	6.7 ± 1.5	a,c
MU	1170.8 ± 97.3	1051.5 ± 70.1	1036.1 ± 103.8	831.6 ± 45.2	a,b,d
Delivery time (min)	6.1 ± 0.6	6.0 ± 0.9	2.2 ± 0.2	2.2 ± 0.4	c,d

a: T-4arc versus T-8arc, b: H-4arc versus H-8arc, c: T-8arc versus H-4arc, d: T-4arc versus H-4arc

According to the results above, of all studied plans T-8arc and H-4arc plans showed the best dosimetry on the respective LINAC. Because of the poor dosimetry, H-8arc was not used for further statistical comparison. Next, H-4arc was statistically analysed with T-4arc and T-8arc, respectively, to reflect the dosimetric differences between the two LINACs.

### Dose comparisons for the plans between the trilogy and the halcyon

The imaging dose of Trilogy is not calculated into the entire plan in contrast to Halcyon plans. In Tables 2 and 3, the doses of PTV and OARs on Trilogy were only caused by the treatment field, without considering the additional effect of the imaging field. For the PTV, a significant difference was observed in the D_mean_ between T-4arc and H-4arc. There were no statistically significant differences in the D_2_, D_98_, V_107_, V_110_, and CI, HI between the two plans. Compared with T-8arc plan, H-4arc significantly increased the D_mean_, D_2_ and V_107_ of the PTV. Better D_98_ and CI were observed in H-4arc. No statistically significant differences were observed in the V_110_ and HI between the T-8arc and H-4arc plans. The results are shown in Table 2.

For the doses of heart and substructures, H-4arc plans increased the V_5_ and D_mean_ of the heart but reduced the V_30_ and V_40_ of the heart and LV (*p* < 0.05). No statistically significant differences were found in the V_10_ and V_20_ of the heart, the V_10_, V_20_ and D_mean_ of the LV, and all dosimetric parameters of the LAD between H-4arc and the two Trilogy plans. H-4arc plans showed the lowest values in the heart and LV doses (V_20_, V_30_, V_40_) and the LAD doses (V_30_, V_40_, D_max_, D_mean_) compared with the two Trilogy plans, but some data showed no statistical significance (Table 3). T-8arc plans significantly reduced the V_5_, V_10_ and D_mean_ of the lungs and the D_mean_ of the liver compared with T-4arc and H-4arc plans, and no statistically significant differences were found in these indexes between the latter two plans. For the V_20_, V_30_, and V_40_ of the lungs, there were no statistically significant differences between H-4arc and the two Trilogy plans (*p* > 0.05). H-4arc plans increased the skin doses (V_30_, V_40_, V_45_, V_50_, and D_mean_) compared with the two Trilogy plans.

T-4arc plans showed the largest number of MUs compared with the T-8arc and H-4arc plans, and no statistically significant difference was found between the latter two plans. The average delivery times of T-4arc, T-8arc, and H-4arc were 6.05 ± 0.57, 6.04 ± 0.89, and 2.18 ± 0.15 min, respectively. H-4arc plans showed the shortest delivery times (Table 3).

## Discussion

In radiotherapy for breast cancer, high position accuracy is essential to prevent under-dose in the target and excessive irradiation to OARs. Image guidance must be taken before each patient is treated on the Halcyon. Compared to two-dimensional (2D) position verification, three-dimensional (3D) position verification like CBCT can more accurately measure 3D vector changes and observe the body position rotation [15, 16]. For breast tissue, CBCT is very useful because it can provide 3D soft tissue and bony anatomy information and can be compared with the planning CT to assess the accuracy of the setting [16, 17]. Rossi et al. [18] reported that CBCT matching is recommended when breast/chest wall patients were treated with the VMAT technique. However, CBCT may be associated with more additional dose and time-consuming than orthogonal planar images. On Halcyon, MV CBCT imaging process take only 15 s. It's beneficial for patients. In C-arm accelerators such as the Trilogy, kilovoltage (KV) CBCT can be used to correct the patient’s position if 3D position verification is necessary. In Halcyon 1.0, MV CBCT was used for image-guided radiotherapy (IGRT). Compared to KV imaging, MV imaging has some advantages, such as identical isocenter as the treatment beam and no metal artifacts. Compared with KV imaging, the main disadvantages of MV imaging are higher dose and lower image quality. About Halcyon’s MV CBCT, Malajovich et al. [19] reported that the highest tissue dose of MV CBCT ranges from 2 to 7 cGy per fraction in different treatment sites, which is equivalent to the fractional dose of KV CBCT during breast and pelvic IGRT application, and MV CBCT images of Halcyon is able to identify different soft tissues and lack of metal-induced artifacts. On Halcyon, two different dose image mode could be selected to apply the CBCT. Compared to the low-dose mode, the high-quality imaging mode does not provide material advantages [19]. We found that high-quality CBCT plans increased the OARs doses compared to low-dose CBCT plans. Therefore, we inferred that low‐dose MV CBCT was the optimal setup-field mode. Flores‐Martinez et al. [6] compared four different setup-field plans for unilateral breast cancer, and in their opinion, low-dose MV CBCT was the most suitable technique for patients treated on the Halcyon. Their results agreed with ours. On Halcyon, the dose of the setup field is incorporated in the calculation of the planned dose, and the irradiated doses to the target and the OARs are also reflected in the total plan dose, which is more intuitive.

Partial-arc plans on the Trilogy showed more dosimetric advantage, especially in low-dose volumes of the heart, left ventricle, and lungs and the mean doses of the heart, lungs, and liver. This is because while designing a partial-arc plan, it is possible to artificially choose the arc degree that irradiates less OAR volume. Rotating the collimator angle and fixing the jaw can further reduce the influence of the leakage between the MLC on the dose of the OARs, which can minimize the dose of the OARs. Boman et al. [11] compared the dosimetric differences between the whole and partial arc VMAT plans of unilateral breast cancer, including regional lymph node irradiation, and the results showed that partial-arc plans significantly reduced the dose of the ipsilateral lung and the V_5_ of the heart but increased the V_5_ of the contralateral breast. The result is similar to ours, but the cases in our study are bilateral breast cancer, which does not involve the dose of the contralateral breast. Comparing the two plans of the Halcyon, the results were contrary to the Trilogy’s, and the whole-arc plans showed better dosimetry. For the PTV, in addition to the mean dose, the whole-arc plan was better than partial-arc plans in terms of the maximum dose, minimum dose, conformity and uniformity. For OARs, partial-arc plans increased the doses to the heart, LV, LAD, and lungs. The results showed that partial-arc plans have no advantage for the Halcyon, which may be related to the jawless setting and the fewer arc degrees. According to the results described above, when designing a treatment plan for bilateral breast cancer, we can choose a more suitable arc setting according to the corresponding LINAC.

Based on the results described above, we mainly compared the dosimetric differences between the Halcyon's whole-arc plan and the Trilogy's two plans. All plans met clinical requirements. For the PTV, apart from the mean dose, there were no significant differences in other dosimetric parameters between the two whole-arc plans on the Halcyon and the Trilogy. Compared with the T-8arc plan, H-4arc showed worse D_mean_, D_2_ and V_107_, and better D_98_ and CI of the PTV. In short, the plans of the two machines were comparable in terms of target dose.

Darby et al. [20] found a linear relationship between the mean dose of the heart and the incidence of ischaemic heart disease, and the incidence increased by 7.4% for each 1-Gy increase in the mean dose. Therefore, the mean dose of the heart is often used as a reference for cardiac toxicity. However, the dose to the cardiac substructure also needs to be considered in radiotherapy. Some studies believe that the LAD and LV are important parts of the heart in the context of radiation-induced heart disease [21–23]. In this study, for low-dose irradiated volumes of the heart and LV, partial-arc plans on the Trilogy showed the lowest values, while for high-dose irradiated volumes, the whole-arc plans on the Halcyon showed the lowest values. For the mean dose of the heart, the partial-arc plans on the Trilogy showed the lowest value, and the whole-arc plans on the Halcyon showed the highest values. This may be related to the additional radiation dose to the heart from each MV CBCT scan. For all dosimetric parameters of the LAD, there is no significant difference between whole-arc plans on the Halcyon and the two plans on the Trilogy.

For lungs, the partial-arc plans on the Trilogy reduced the low-dose irradiated volume (V_5_, V_10_) and the mean dose but increased the high-dose irradiated volume (V_40_). There was no significant difference in the comparison of the V_20_ and V_30_ in the lungs between all plans of the two LINACs. For all dosimetric parameters of the lungs, there was no significant difference between the two whole-arc plans on the two LINACs. Fiorentino et al. [24] retrospectively analysed the VMAT plans of 16 patients with bilateral breast cancer. For lungs, the average values of the D_mean_, V_5_ and V_20_ were 11.8 ± 2.3 Gy, 78.9 ± 15.3% and 15.7 ± 5%, respectively. No acute and late complications above grade 2 were observed during the 24 months of follow-up. In our study, the mean dose, V_5_ and V_20_ of the lungs in all plans were lower than in their study.

The plans on the Halcyon increased the skin’s dose compared to the two plans on the Trilogy. O’Grady et al. [25] found that 6 X FFF fields on the Halcyon increased the superficial dose compared to FF fields for breast cancer radiotherapy, as demonstrated by in vivo measurements, phantom measurements, and planning comparisons. Their results were the same as ours. Because the rays are softened after the flattening filter is removed, the 6 MV X-rays in FFF mode are equivalent to lower-energy rays, resulting in a shallower depth of the dose build-up area, thereby increasing the superficial dose. Studies on the Monte Carlo (MC) have shown that the influence of contamination electrons in the FFF mode is greater, which further leads to an increase in surface dose [26, 27]. Barsky et al. [5] retrospectively analysed 34 breast cancer cases treated on the Halcyon, and the results showed that breast cancer cases were well tolerated on Halcyon; additionally, the acute toxicity was comparable to the published reports using conventional LINACs. The difference from our study is that they used tangential fields instead of the VMAT in our study. On the Halcyon, the effect of VMAT on the skin dose needs more prospective clinical studies to be proved.

In contrast to Halcyon, KV CBCT could be used for image-guided on Trilogy. Although the dose of KV CBCT is less than that of MV CBCT, it is not negligible. The difference with Halcyon is that the imaging dose of Trilogy is not calculated into the entire plan. So, in fact, the dose difference between the two machines may vary slightly. The whole-arc plans on Halcyon reduced the number of MUs compared to whole-arc plans on Trilogy and showed similar MUs compared to partial-arc plans on Trilogy. On Halcyon, the imaging dose is integrated into plans and thus it is contributing to the PTV and OAR dose. However, in Trilogy the imaging dose is not accounted for plans, what might explain the lower MUs required on Halcyon.

Previous studies have demonstrated that the Halcyon could reduce the treatment time significantly compared with conventional LINACs [3–7]. Our study proved the same results in radiotherapy for bilateral breast cancer. This may be related to the Halcyon's faster gantry and MLC speed and higher dose rate. The shortening of the treatment time can reduce intra-fraction movement, improve patient comfort, and increase machine throughput.

An issue that needs to be addressed is the correction of rotation errors. On C-arm accelerators like Trilogy, we can use the six degree of freedom (6-DoF) couch to correct the rotation errors. The combination of 6-DoF couch and CBCT can correct the translational and rotational setup errors and improve the positioning accuracy obviously [28, 29]. However, on O-ring accelerators like Halcyon, the installed 3-DoF couch allows only for correction of translational shifts. On the Halcyon, if large rotation errors are found, time-consuming repositioning may be required to improve the positioning accuracy. This drawback might significantly compromise the advantage of faster treatment plan delivery with Halcyon compared to Trilogy. Therefore, in order to reduce the positioning time and improve the positioning accuracy, it is necessary to improve the immobilization equipment or adopt online 3D IGRT equipment, such as surface imaging. 3D surface imaging system is a quick and non-invasive method to assist the patient in setting up, which could improve the accuracy and speed of patient positioning in breast cancer radiotherapy [30, 31]. On Halcyon, 3D surface imaging is especially suitable, which could correct rotation errors in patient positioning and monitor patient movement during beam delivery [32]. The combination of 3D surface imaging and daily CBCT may greatly improve treatment accuracy and reduce positioning time, which needs more research to prove in the future.

Many studies [33–35] have shown that deep inspiration breath hold (DIBH) can significantly reduce the radiation dose to the heart and lungs for breast cancer radiotherapy. However, the technique requires patients to hold their breath for a certain amount of time. Minimizing the treatment time is beneficial to patients with DIBH. The combination of Halcyon and VMAT can further reduce the treatment time and expand the application range of DIBH. The robustness of VMAT plans under respiratory motion or other influences which might change form or position of PTV needs to be considered. Field-in-field and fixed-field intensity-modulated radiation therapy can reduce the influence of respiratory movement and skin deformation or swelling during treatment by opening the MLC outside the skin or adding skin flash. When designing VMAT plans, we could increase the robustness of the plan by adding a virtual bolus on the skin [11, 36, 37].

## Conclusions

VMAT plans on the Halcyon can meet the clinical requirements in radiotherapy for bilateral breast cancer. Low-dose MV CBCT can be selected as an optimal setup mode. For Halcyon, the whole-arc plans are better than the partial-arc plans. While the Halcyon increases the mean dose to the heart and the dose to the skin compared with the conventional LINAC, it is comparable to the Trilogy in doses to the cardiac substructure and other OARs and significantly reduces the delivery time.

## Data Availability

The data are available upon request.

## Abbreviations

LINAC: Linear accelerator
FFF: Flattening-filter-free
MLC: Multi-leaf collimator
IGRT: Image guided radiotherapy
VMAT: Volumetric modulated arc therapy
MV CBCT: Megavoltage cone beam computed tomography
MUs: Monitor units
CTV: Clinical target volume
PTV: Planning target volume
OARs: Organs at risk
LV: Left ventricle
LAD: Left anterior descending artery
B-P: Body minus the PTV
DMI: Digital megavoltage imaging
AAA: Analytic anisotropic algorithm
CI: Conformity index
HI: Homogeneity index
ANOVA: One-way analysis of variance
CBCT-H: Plan with high-quality MV CBCT
CBCT-L: Plan with low-dose MV CBCT
MV-H: Plan with high-quality MV pair
MV-L: Plan with low-dose MV pair
T-4arc: Whole-arc plans on Trilogy
T-8arc: Partial-arc plans on Trilogy
H-4arc: Whole-arc plans on Halcyon
H-8arc: Partial-arc plans on Halcyon
MC: Monte Carlo
DIBH: Deep inspiration breath hold
